# Characterization and genomic analysis of *Bacillus megaterium* with the ability to degrade aflatoxin B_1_

**DOI:** 10.3389/fmicb.2024.1407270

**Published:** 2024-08-07

**Authors:** Ting Li, Xiaoxi Chang, Zixuan Qiao, Guangxi Ren, Na Zhou, Jiaxin Chen, Dan Jiang, Chunsheng Liu

**Affiliations:** ^1^School of Chinese Medicine, Beijing University of Chinese Medicine, Beijing, China; ^2^Shaanxi University of Chinese Medicine, Co-Construct Collaborat Innovat Ctr Chinese Medicine Research, Xianyang, China

**Keywords:** aflatoxin B_1_, *Bacillus megaterium*, coix seed, fermentation supernatant, whole-genome sequencing

## Abstract

Coix seed is a good product for both medicinal and food use, which is highly susceptible to aflatoxin B_1_ (AFB_1_) contamination during field transport, storage, and processing. The aim of this study is to find microbial strains that can solve the problem of contamination of coix seed. In this study, the AFB_1_-degrading microorganism SX1-1 was isolated and identified as a *Bacillus megaterium* based on morphology, microscopy, and 16S rDNA sequencing. The optimum culture conditions for SX1-1 to degrade AFB_1_ were determined to be 12 h. The optimum degradation conditions were 72 h, 57°C, and an initial pH of 8.0. The highest degradation of AFB_1_ was observed in the fermentation supernatant of the SX1-1 strain, with a degradation rate of 97.45%. In addition, whole-genome sequencing analysis of this strain revealed the presence of a number of enzymes that could potentially degrade AFB_1_. Importantly, SX1-1 was able to degrade AFB_1_-contaminated coix seed *in situ* by 50.06% after co-culture. In conclusion, this strain had a high AFB_1_ degradation ability, and has great potential and great application as a biocontrol agent for AFB_1_ degradation of coix seed.

## 1 Introduction

*Aspergillus flavus* is a common fungus that is highly vulnerable to infection during cultivation, transport, and storage of food and herbal medicines (Qin et al., 2021; Li et al., 2021b). Aflatoxins are mainly secondary metabolites produced by *Aspergillus flavus* and *Aspergillus parasiticus*. Among the currently identified aflatoxin structures, AFB_1_ is extremely carcinogenic and highly toxic (Zhou et al., 2021), and has been classified as a Group I carcinogen by the International Agency for Research on Cancer (Luo et al., 2021). AFB_1_ is a derivative of dihydrofuran coumarin in chemical structure, consisting of a difuran ring, an oxonaphthalone (coumarin), and a pentane ring. The double bond located at positions 8 and 9 of the furan ring and the lactone ring in the coumarin structure are often closely related to the strong toxicity of AFB_1_ (Bedard and Massey, 2006; Xu et al., 2023a; Arimboor, 2024; Tang et al., 2024). Aflatoxins are of great concern due to their extremely high toxicity and people worldwide are at risk from aflatoxins through food, herbs, etc. (Hamad et al., 2023). The current potential for aflatoxin production in food and herbs during transport and storage continues to increase. Therefore, there is an urgent need to address aflatoxin contamination in a scientific and efficient manner to ensure the safety of both food and herbal medicines.

Coix seed is the dried mature seed kernel of the grass plant *Coix lacryma-jobi* L. var. *mayuen* (Roman.) Stapf, which is a typical representative of the dual-use of medicine and food, and has important medical and edible values. However, being rich in starch and oil, coix seeds are susceptible to contamination by AFB_1_. Several studies have confirmed the presence and severity of AFB_1_ contamination in coix seeds (Zhou et al., 2023). The Chinese Pharmacopeia standard for AFB_1_ in coix seeds has been revised to the maximum limit for the prevention and control of AFB_1_ contamination in coix seeds, which should not exceed 5 μg of AFB_1_ per kilogram of coix seeds. Therefore, the control of AFB_1_ contamination in coix seeds is of great importance to ensure the safety of the use of coix seeds.

Removal of AFB_1_ is particularly important. Current AFB_1_ removal methods are mainly physical, chemical, and biological. The main physical methods include adsorbents (Zhang et al., 2021a), UV irradiation (Sun et al., 2021), roasting, ionizing radiation, etc. Chemical methods include alkaline and oxidation methods, but both methods have varying effects on the quality of food and herbs, and are prone to chemical residues and high equipment costs (Liu et al., 2022). In contrast, biological methods have the advantages of high specificity, high detoxification efficiency, low environmental pollution, and low impact on the quality of food and herbal medicines (Xue et al., 2023). Therefore, biological strategies are considered as a promising approach to address the problem of AFB_1_ contamination. A number of organisms have now been tested for their ability to degrade aflatoxin. These include *Streptomyces* (Campos-Avelar et al., 2021), *Lactobacillus* (Pakizeh et al., 2022), *Bacillus* (Li et al., 2021a), *Bjerkandera adusta* (Choo et al., 2021), edible fungi (Guo et al., 2024), etc. In many cases, these strains can reduce the content of AFB_1_ through cellular adsorption or degrade AFB_1_ through metabolites produced by the cells. However, due to the narrow spectrum of AFB_1_-degrading strains and their poor suitability, this area of research has not been sufficiently developed and good strain resources are still urgently needed.

The mechanisms of microbial degradation of AFB_1_ are complex. Many efforts have been made to elucidate the mechanisms of microbial degradation of AFB_1_. The application of genomic technologies is an important technical tool to unravel the complex mechanisms of AFB_1_ degradation by microorganisms. Previous literature suggests that genomes have been used to discover AFB_1_-degrading enzymes in *Bacillus licheniformis* and *Aspergillus niger*, which supports the use of genomic techniques to elucidate microbial degradation of AFB_1_ (Fang et al., 2020). Therefore, in this study, we first screened for efficient AFB_1_-degrading microorganisms and analyzed their potential mechanisms from their genomes, the degradation strategy of highly efficient AFB_1_-degrading bacteria on food and also investigated using AFB_1_ impaired coix seeds as a substrate. This study aims to provide effective strains to solve the problem of AFB_1_ contamination in food or medical materials.

## 2 Materials and methods

### 2.1 Chemicals and sampling

Aflatoxin standards were purchased from the Food and Drug Administration (Lot number: 61001-201703, Beijing, China). Coumarin medium (magnesium sulfate 0.25 g, potassium dihydrogen phosphate 0.25 g, ammonium sulfate 0.5 g, potassium nitrate 0.5 g, calcium chloride 0.05 g, agar 20 g, coumarin 1 g, and deionized water 1 L) and LB solid medium (tryptone 10 g, yeast extract 5 g, sodium chloride 10 g, agar 20 g, and deionized water 1 L) were self-prepared.

Based on previous reports, we found that the changes of *Aspergillus flavus* in Polygalae radix from different regions in China showed regularity (Chang et al., 2021). There may be microorganisms in Polygalae radix that can inhibit *Aspergillus flavus*, and even have potential activity in degrading AFB_1_. In addition, few studies have isolated strains that degrade AFB_1_ from Polygalae radix. Therefore, we isolated potential functional bacteria from the surface of Polygalae radix from different regions. These samples of Polygalae radix came from Yuncheng, Shanxi province, China (seven samples), Xingtai, Hebei province, China (one sample), Weinan, Shaanxi province, China (one sample), Luoyang, Henan province, China (one sample), Chifeng, Inner Mongolia, China (one sample), and Bijie, Guizhou province, China (one sample), respectively.

### 2.2 Preliminary screening of AFB_1_ degrading strains

Aflatoxin B_1_-degrading strains were isolated from the surface of the Polygalae radix of various origins. Specifically, 1 g of Polygalae radix was placed in a sterile centrifuge tube, 9 mL of sterile water was added, vortexed and homogenized, and 500 μL of it was evenly coated in coumarin medium, with an equal amount of sterile water as a control, and incubated for 72 h at 37°C. Coumarin medium was used to screen for AFB_1_ degrading strains (Tang et al., 2024). Coumarin was the only carbon source and the strains that could grow in coumarin medium were considered to be able to degrade AFB_1_. These bacterial strains grown in coumarin medium were purified to single colonies using LB solid medium (tryptone 10 g, yeast extract 5 g, sodium chloride 10 g, agar 20 g, deionized water 1 L) and preserved.

### 2.3 Re-screening of AFB_1_ degrading strains

The bacterial strains grown in coumarin medium were re-screened. The purified bacterial strains were inoculated into 50 mL of liquid LB medium and incubated for 48 h at 160 rpm at 37°C. The culture broth was collected by centrifugation at 8,000 rpm for 15 min at 4°C. 10 μL of AFB_1_ standard was added to 490 μL culture broth for the experimental group. The same amount of culture broth was used as the control group, and culture broth containing the same amount of methanol solution was used as the blank group. All treatments were incubated for 72 h and the reaction was stopped by adding 500 μL of methanol. After incubation, the reaction was centrifuged at 12,000 rpm for 30 min and analyzed by UPLC–MS. The degradation rate of AFB_1_ was calculated as follows: Degradation rate (%) = (1 − AFB_1experimental group_/AFB_1control group_) × 100%.

### 2.4 Identification of SX1-1

The screened bacterial strain was designated SX1-1. Gram staining, colony morphology, size, and acid and salt tolerance were observed, and starch hydrolysis, lipid hydrolysis and glucose utilization, indole production, and methyl red reactions were determined. Identification was also carried out by 16S rDNA sequence analysis. Universal primers 27F (AGAGTTTGATCCTGGCTCAG) and 1492R (GGTTACCTTGTTACGACTT) were used for PCR amplification of the strains under investigation. A amplification products were analyzed by 1.0% agarose gel electrophoresis and products were sequenced by Sangon Biotech (Shanghai) Co., Ltd. The sequencing results were proofread and spliced using ContigExpress software, and the resulting sequences were used to build a clustering tree based on neighbor-joining (NJ) using MEGA 6.0 software (molecular evolutionary genetics analysis), and the support test for each branch was performed by bootstrap (1000 repetitions) to test the support of each branch.

### 2.5 Determination of the active fraction of AFB_1_ degradation by SX1-1

SX1-1 culture with OD_600nm_ = 1.0 was inoculated into fresh LB medium and incubated for 12 h. The bacterial culture was then divided into two tubes and centrifuged at 12,000 rpm at 4°C for 10 min to separate the supernatant and the sediment. The supernatant of one tube was filtered by a 0.22 μm water filtration membrane to obtain the fermentation supernatant sample. The sediment was washed three times with phosphate balanced solution (PBS) (0.1 M, pH 7.0) buffer and then resuspended in PBS as the active cell solution. The sediment in the other tube were obtained as described above and were subsequently treated by ultrasonic treatment (400 W, 20–25 kHz) for 15 min, centrifugation was then carried out at 12,000 rpm at 4°C for 10 min and the supernatant was passed through a 0.22 μm filtration membrane as the cell lysate (Deng et al., 2022). 490 μL of supernatant, active cell solution and cell lysate were taken separately, 10 μL of AFB_1_ solution was added and incubated at 37°C for 72 h. The toxin residues were measured in the control group with equal amounts of PBS solution added to equal amounts of AFB_1_ solution and a blank group with LB medium without any treatment.

Another supernatant was taken and treated with 1 mg·mL^−1^ proteinase K and heat treatment, and the untreated supernatant was used as control (Chen et al., 2022; Deng et al., 2022) 490 μL of the each group were taken and added to 10 μL of AFB_1_ solution at a concentration of 0.005 μg·mL^−1^, and incubated at 37°C for 72 h under protected from light. The reaction was terminated with 0.5 mL of methanol and the untreated LB medium was used as the blank group to measure the toxin residue. All experiments were repeated three times.

### 2.6 SX1-1 kinetic study

SX1-1 cultures with OD_600nm_ = 1.0 were inoculated into fresh LB medium and sampled at 0, 12, 24, 48, and 72 h under the same conditions. The samples were centrifuged at 10,000 rpm for 10 min to extract the supernatant and the biomass of the flora was measured by OD_600nm_ and the free protein content was measured using the BCA kit (R23183, Shanghai Yuanye Biotechnology Co., Ltd.). To assess the effect of treatment time, the culture systems were sampled at 0, 12, 24, 48, 72, and 96 h to determine the AFB_1_ residue. To assess the effect of treatment temperature, culture systems were incubated in the dark at 27, 37, 47, 57, and 67°C and AFB_1_ levels were determined. To evaluate the effect of pH, the initial pH of the incubation system was adjusted to 5.0, 6.0, 7.0, 8.0, and 9.0 with sterile 0.1 mol·L^−1^ HCl solution or 0.1 mol·L^−1^ NaOH solution, respectively, and the reactions were carried out at the optimum time and temperature to investigate the effect of pH on the degradation process.

### 2.7 UPLC–MS analytical conditions

The instrument was methodologically tested before use and samples were determined after passing the test. The chromatographic conditions were as follows: column BEH C18 (100 mm × 2.1 mm, 1.7 μm, Waters, United States), mobile phase 0.1% formic acid in water (A) and methanol (B), separation conditions as follows: 0 min, 90% A, 0.5 min, 90% A, 6 min, 10% A, 7 min,10% A, 9 min, 90% A, and 10 min, 90% A. The sample volume was 2 μL. Samples were detected by triple quadrupole mass spectrometry using an electrospray ionization (ESI) source in positive ion mode with an AFB_1_ parent ion of (m/z) 313.2, a quantitative daughter ion of (m/z) 241.1, a collision voltage of 36 V and a daughter ion of 285.1, and a collision voltage of 24 V.

### 2.8 Whole genome sequencing of SX1-1

SX1-1 cultures with OD_600nm_ = 1.0 were inoculated into fresh LB medium, the bacterial broth was centrifuged at 4,000 × *g* for 10 min and the organisms were washed twice with sterile water. Genomic DNA was extracted by the SDS method, and DNA purity and integrity were checked by 1.0% agarose gel electrophoresis and quantified by Qubit. Qualified DNA samples were randomly fragmented into approximately 350 bp fragments using a Covaris ultrasonic fragmentation machine and genomic libraries were prepared using the NEBNext®Ultra™ DNA Library Prep Kitfor Illumina (NEB) kit. Sequencing was then performed on the Illumina NovaSeq PE150.

### 2.9 Genome annotation and analysis

The sequencing results were assembled and corrected to ensure the quality of the genome. Genewise software (version 2.4.1) was used for coding gene prediction, and the protein sequences of the predicted genes were compared with each functional database by Diamond (evalue ≤1e^−5^), and for each sequence (Chen et al., 2022), the comparison result with the highest score (default identity ≥40%, coverage ≥40%) was annotated. The main common functional databases currently available for annotation were Gene Ontology (GO), Clusterof Orthologous Groups of proteins (COG) (Tatusov et al., 2003), Kyoto Encyclopedia of Genes and Genomes (KEGG), Carbohydrate Active enZYmes DatabaseS (CAZy), Virulence Factor Database for Pathogenic Bacteria (VFDB), etc.

### 2.10 *In situ* degradation of AFB_1_ contaminated coix seeds by SX1-1

We collected coix seeds of different origins from the Chinese herbal market, stored them at 25°C, and tested the AFB_1_ content according to the above UPLC-MS analytical conditions every half month until the AFB_1_ content exceeded the limit (5 μg·kg^−1^) specified in the Chinese Pharmacopeia. We used the coix seeds with AFB1 content exceeding the limit as our subsequent raw material for *in situ* degradation.

12 g of AFB_1_-exceeding coix seeds was weighed, 40 mL of fermentation supernatant of SX1-1 strain (OD_600nm_ = 1.0, pH 8.0) was added to coix seeds for incubation as experimental group, and an equal amount of LB liquid medium was added as control group, which was combined with the actual samples stored in the selected samples to be incubated at 37°C for 72 h. At the end of the incubation, the liquid was discarded, and the coix seeds was dried, pulverized, and extracted for the determination of the content of AFB_1_ contained in the coix seeds.

### 2.11 Data analysis

All experiments were repeated three times and data were expressed as mean ± standard deviation (RSD) and analyzed using one-way ANOVA or Tukey’s test (SPSS 22.0), *p* < 0.05 was considered to be statistically significant.

## 3 Results and discussion

### 3.1 Screening and identification for AFB_1_-degrading bacteria SX1-1

**Figure 1:**
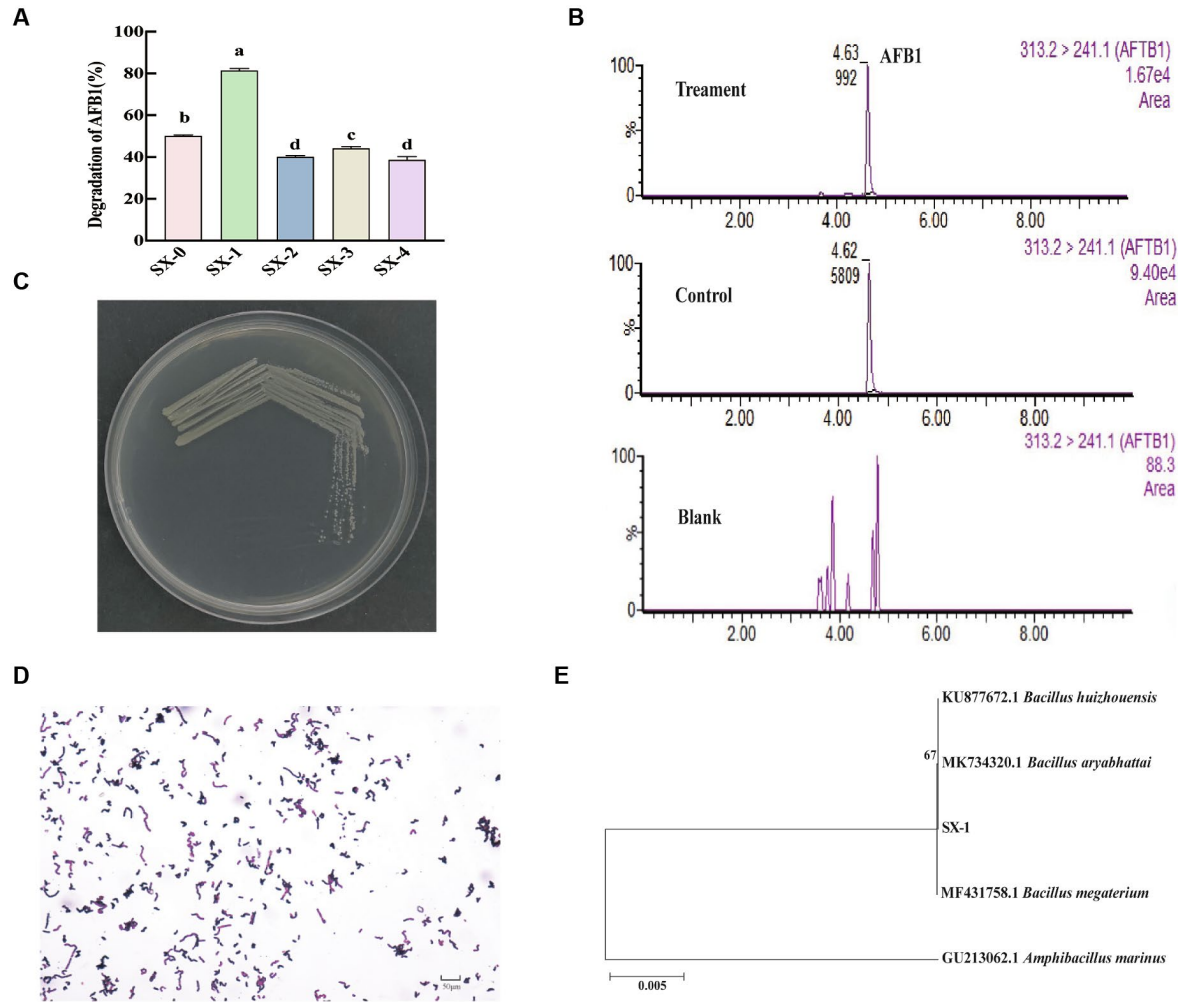
Screening and identification of AFB_1_-degrading strains (**A**: AFB_1_-degrading bacteria were incubated in liquid medium with coumarin as the sole carbon source for 72 h at 37°C. These values are the mean ± standard deviation of the three replicate groups; different letters indicate significant differences, *p* < 0.05. **B**: UPLC-MS mass spectra of the treated, control and blank groups, respectively, with the positions of the absorption peaks labeled with retention time and peak area, unlabeled indicates low concentration and not reaching the detection limit; **C**: state of SX1-1 cultured on LB plate; **D**: state of SX1-1 under 40x microscope after Gram staining; **E**: N-J tree of SX1-1, SX1-1 were grouped together with *Bacillus aryabhattai*, *Bacillus megaterium*, and B*acillus huizhouensis*).

The molecular structure of aflatoxin, which has a coumarin ring as its parent nucleus, is very stable, and thus bacteria that can grow on coumarin as the sole carbon source are expected to degrade and utilize AFB_1_. In a preliminary screening of degrading bacteria, we obtained 55 strains capable of growing in plates with coumarin as the sole carbon source. The ability of the strains to degrade AFB_1_ was further confirmed using UPLC-MS. The results showed that five strains had good degradation activity against AFB_1_ (Figure 1A). The degradation ability of strain SX1-1 was significantly higher than other strains, with a degradation rate of 81.34%. Meanwhile, the confirmation results of UPLC-MS spectra further showed that the typical absorption peak of AFB_1_ appeared at 4.6 min in the AFB_1_ control solution, and that of the control also appeared around 4.6 min, while the peak area and peak height of the sample were significantly lower than those of the control (Figure 1B), indicating the efficient removal ability of SX1-1 for AFB_1_. This was the highest degradation capacity of AFB_1_ among the *Bacillus megaterium* reported to our knowledge (Liu R., et al., 2013).

Further identification of this SX1-1 was required for the effective use of this strain. Colonies of SX1-1 on LB plates were creamy white, round colonies with clean and thin edges and a slightly raised, bumped center (Figure 1C). Under the microscope, the cell morphology was rod-shaped and positive to Gram staining (Figure 1D). Sequences of approximately 1,500 bp were obtained by amplification and sequencing with 16S rDNA universal primers. Homology analysis of the sequenced PCR products showed that the sequences of SX1-1 were grouped together with *Bacillus aryabhattai*, *Bacillus megaterium*, and *Bacillus huizhouensis* (Figure 1E). This suggests that these strains may be more conservative in the primer fragments we used. Based on the biochemical reaction characteristics (Supplementary Table S1), combined with morphological and phylogenetic features, we finally identified SX1-1 as *Bacillus megaterium*.

### 3.2 Determination of the active fractions of the strain SX1-1 for AFB_1_ degradation

**Figure 2:**
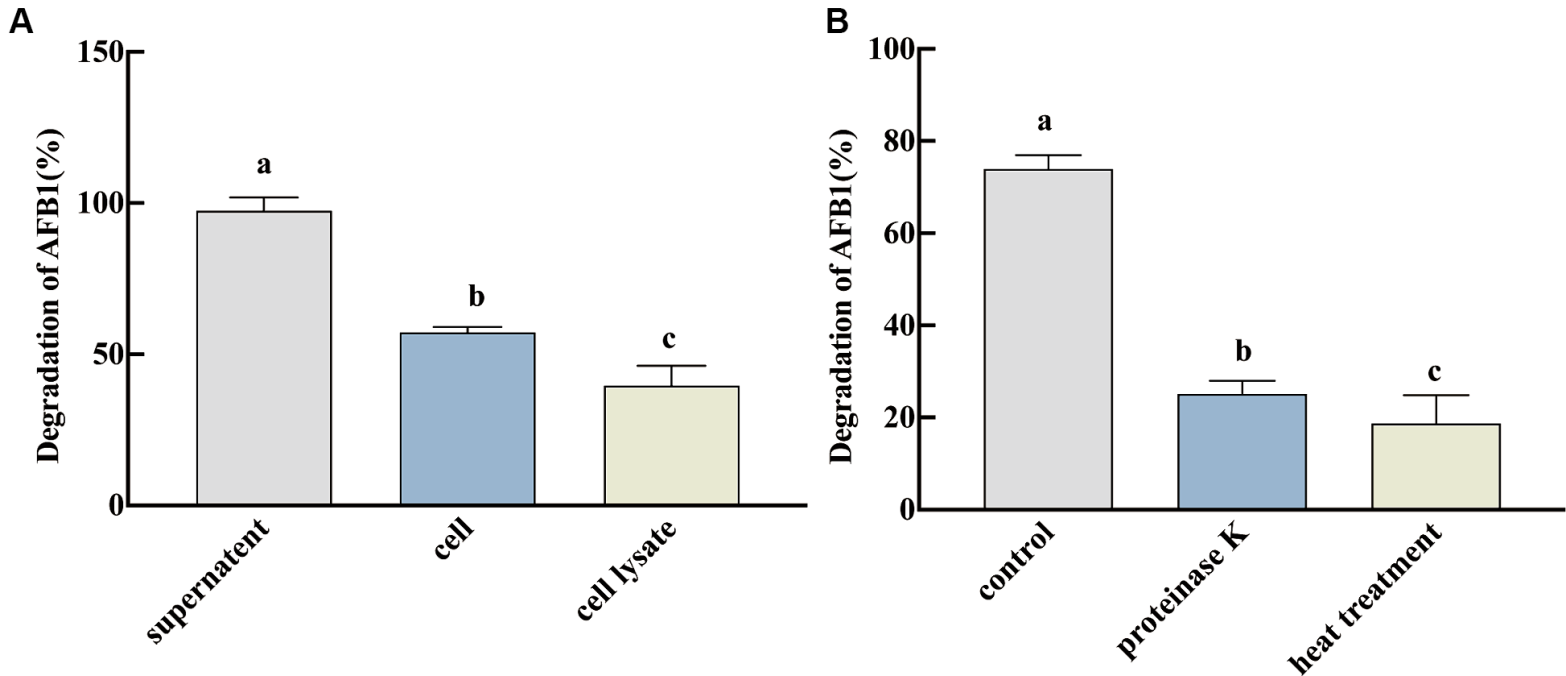
Degradation of AFB_1_ by active fractions of SX1-1 (**A**: degradation of AFB_1_ by fermentation supernatant, active cell solution, and cell lysate after 72 h incubation with a certain concentration of AFB_1_; **B**: degradation of AFB_1_ by proteinase k of fermentation supernatant and by fermentation supernatant after heat treatment and 72 h incubation with a certain concentration of AFB_1_). Different letters indicate significant differences, Tukey’s test, *p* < 0.05.

The ability of the different fractions of SX1-1 (fermentation supernatant, cell lysate, and active cell solution) to degrade AFB_1_ was further evaluated. It was found that the degradation efficiency of the fermentation supernatant was significantly higher than that of the active cell solution and cell lysate at 72 h, with a degradation rate of 97.45% (Figure 2A). This result indicated that the removal of AFB_1_ by SX1-1 was mainly dependent on the fermentation supernatant, similar to previous studies. To further investigate whether the fermentation supernatant degraded AFB_1_ as a protein or an enzyme, we subjected the fermentation supernatant to proteinase K and heat treatment. It was found that the degradation rate of AFB_1_ in SX1-1 fermentation broth after proteinase K treatment (degradation rate was 25.17%) and after heat treatment (degradation rate was 18.72%) was significantly low than in the control group (Figure 2B). Since Proteinase K was a potent proteolytic enzyme and heating can also denature and inactivate proteins, although it is also possible that a small amount of SX1-1 is adsorbed to the cell membrane, we speculate that extracellular enzymes or proteins in the supernatant of SX1-1 fermentation are still involved in the degradation of AFB_1_ as a major fraction.

### 3.3 Kinetic studies of SX1-1

**Figure 3:**
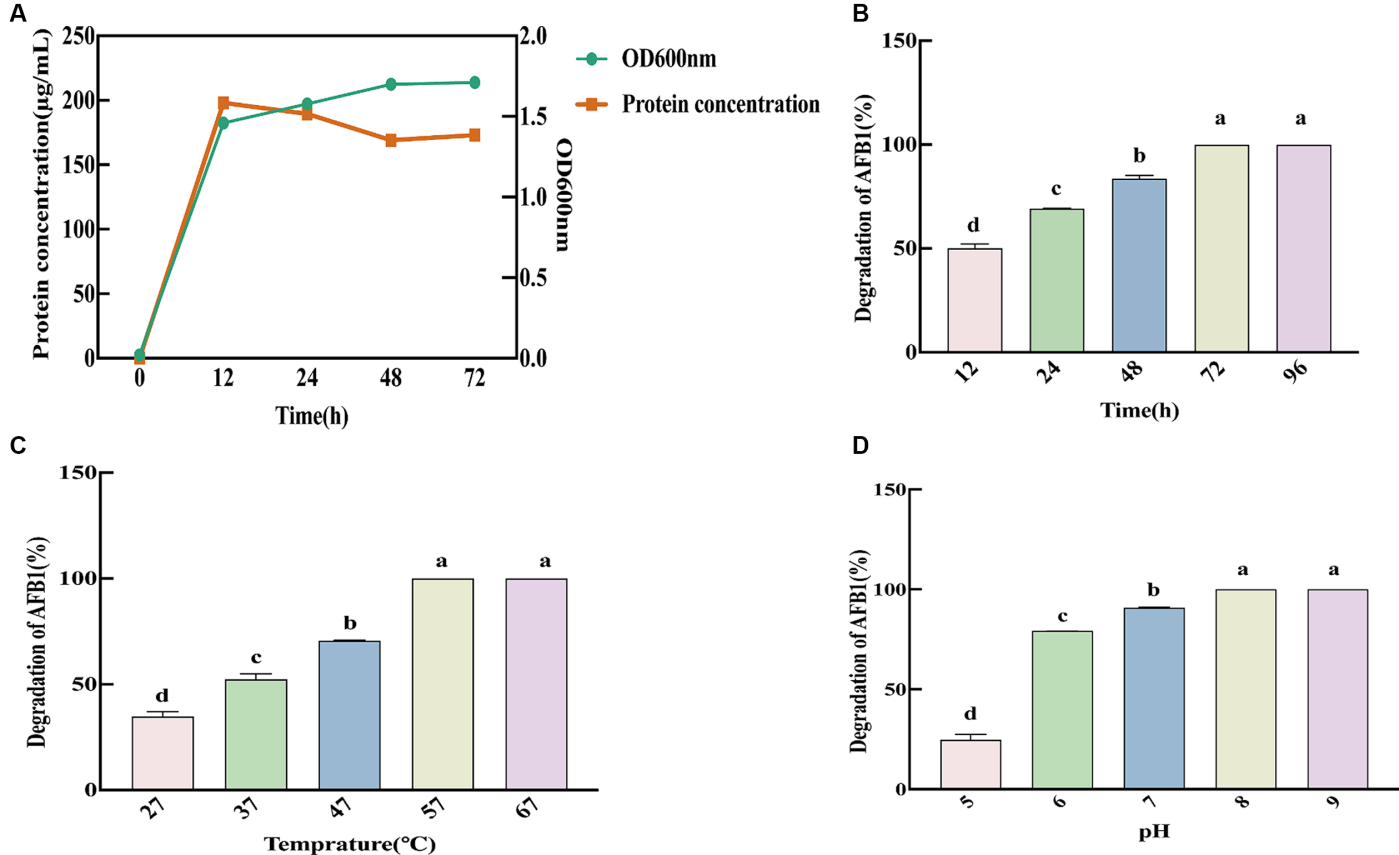
Kinetics of SX1-1 (**A**: optimal incubation time of SX1-1 as reflected by OD_600 nm_ and protein concentration; **B**: different incubation times on SX1-1 fermentation supernatant-mediated AFB_1_ degradation; **C**: different incubation temperatures on SX1-1 fermentation supernatant-mediated AFB_1_ degradation; **D**: different initial pH on SX1-1 fermentation supernatant-mediated AFB_1_ degradation; Different letters indicate significant differences, Tukey’s test, *p* < 0.05).

We measured the OD_600nm_ and free protein content of SX1-1 for 72 h after inoculation into LB (Figure 3A), and the results showed that the strain multiplied rapidly within 12 h, with a large increase in biomass. Therefore, 12 h was considered as the optimal incubation time for SX1-1, when the OD_600nm_ was 1.46 and the protein content was 198.11 μg·mL^−1^. In addition, pH, temperature, and incubation time may affect the ability of SX1-1 fermentation supernatant to degrade AFB_1_. The results of the degradation ability of SX1-1 fermentation supernatant after coincubation with AFB_1_ for 96 h showed that the degradation ability of the strain reached its optimum after 72 h and remained stable (Figure 3B). As the incubation temperature increased, the degradation rate of AFB_1_ by SX1-1 gradually increased, and AFB_1_ could be completely degraded at 57°C and beyond (Figure 3C). As the initial pH increased, the degradation rate of SX1-1 gradually increased, with complete degradation at pH 8 (Figure 3D), indicating that the degradation of AFB_1_ by SX1-1 was more favorable under alkaline conditions. We therefore founded that the optimum incubation conditions for SX1-1 were 12 h and the optimum degradation conditions were 72 h, 57°C and an initial pH of 8.0. These results indicated that SX1-1 was more stable and would increase the practical application of SX1-1 in the future.

### 3.4 Genomic analysis of SX1-1

**Figure 4:**
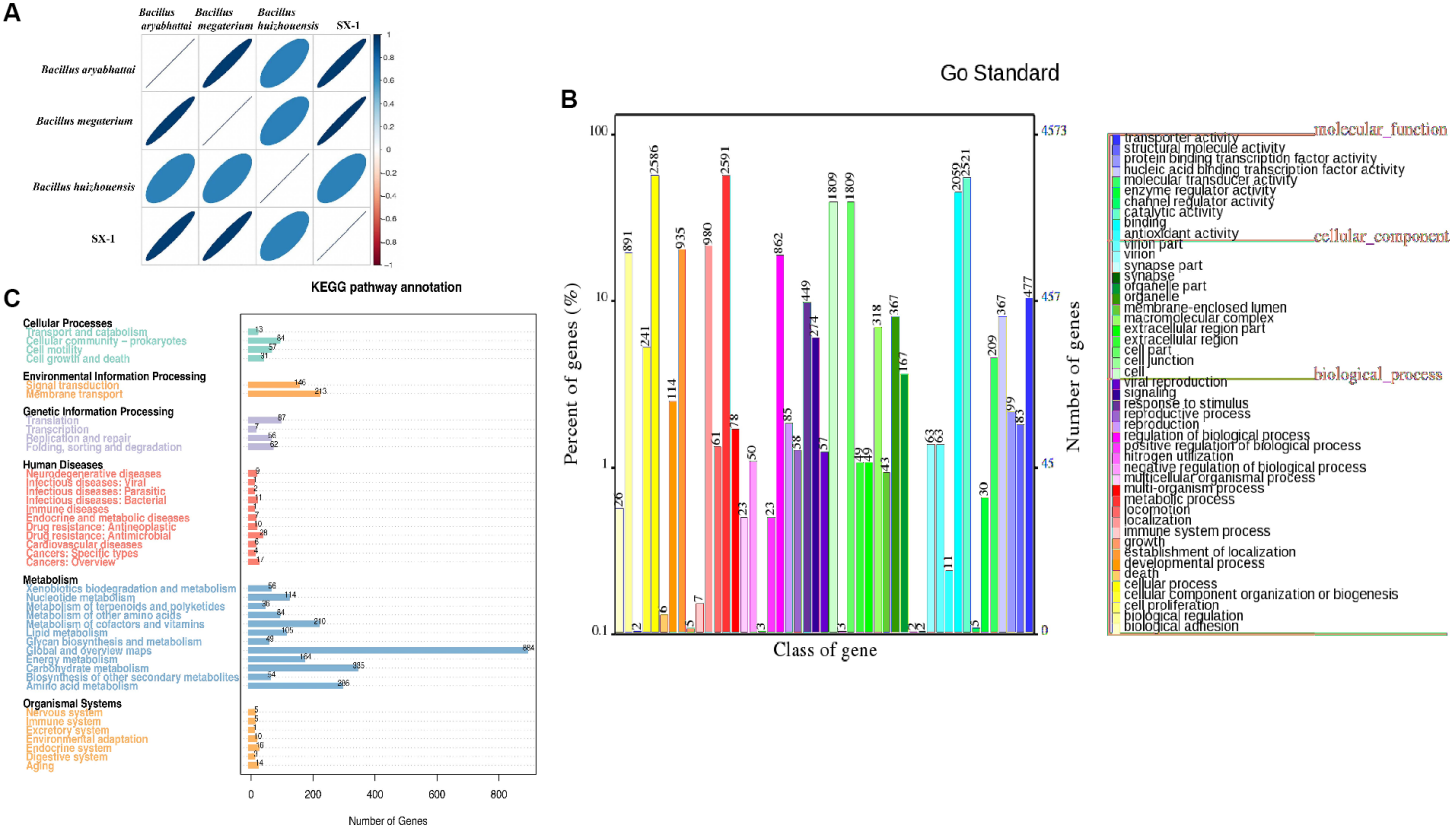
Functional gene analysis of SX1-1 genome sequencing and annotations from various databases (**A**: heatmap of ANI values between samples, different colors represent high and low ANI values; the relationship between ANI values and colors is illustrated in the legend on the right, darker colors represent larger ANI values between samples; **B**: functional classification of genes obtained from GO database annotations; **C**: KEGG metabolic pathway of the genome classification diagram).

We sequenced the genome of SX1-1 to investigate its genomic properties that cause AFB_1_ degradation. High quality data of 1,006 Mb was obtained for the SX1-1 genome, with an estimated genome size of 5.41 Mb, 201 contigs larger than 500 bp, a value of 370,411 for N50 and an average GC content of 37.1%. The genome contained 8,633 genes, 7,059 coding genes, and 147 non-coding RNAs, including 127 tRNAs, 13 rRNAs (115 srRNAs, 116 srRNA, and 123 srRNA) and seven sRNAs. In the ANI analysis, SX1-1 had a matrix value of 97.16% (over 95% considered to be the same species) with *Bacillus megaterium* (Figure 4A), confirming it as *Bacillus megaterium*.

The annotations in the GO database (Figure 4B), where genes were assigned functions, were divided into three main categories, including cellular component, molecular function, and biological process, with the largest number of genes involved in metabolic processes being annotated in biological process (2,591). In the molecular function category, the largest number of genes involved in catalytic reactions was 2,521, and in the cellular component category, the largest number of genes related to cells and cellular components was 1,809, respectively. Overall, the number of genes involved in bacterial metabolic processes and cellular processes was the highest. This implied the presence of a large number of metabolic and catalytic genes in SX1-1, suggesting a strong metabolic capacity and possible involvement in enzymatic catalysis. The functions annotated in the KEGG database were consistent (Figure 4C). The genes involved in carbohydrate metabolism, amino acid transport, and membrane transport pathways were the most abundant, with 335, 286, and 213 genes, respectively. We then analyzed the secondary metabolite gene clusters using antiSMASH, and found 11 biosynthetic gene clusters in the SX1-1 (Supplementary Figure S1). There were three gene clusters for terpene synthesis and the number of annotated genes was 65. These BGCs may contribute to the survival and better functioning of SX1-1.

Enzymatic reactions were considered to be the main mechanism for microorganisms degradation of AFB_1_ (Watanakij et al., 2020; Zhao et al., 2020; Zhang et al., 2021b). We further mined the enzymes annotated in the SX1-1 genome and showed that more than 27% of the enzymes were annotated to the glycoside hydrolase (GH) family (56 genes), 31.7% to the CBM family (65 genes), 30.7% to the GT family (63 genes), and 9.7% to the CE family (20 genes). Further analysis revealed the presence of alkaline phosphatase, peroxidase, superoxide dismutase, and laccase in the COG database (Supplementary Table S2). Enzymes such as laccase (Sun et al., 2023), manganese peroxidase (Xu et al., 2023), and aflatoxin oxidase (Deng et al., 2022) were reported to be the main enzymes involved in the degradation of AFB_1_, among which laccase, a class of polyketide oxidases, is considered as a green tool for xenobiotic degradation, which converts AFB_1_ into less toxic compounds through 3-R-hydroxylation. The abundant presence of these enzymes suggests that they may be responsible for the effective degradation of AFB_1_ by SX1-1. However, since the structure of aflatoxin B_1_ includes phenolic, keto, alcohol, and ether groups, it is reasonable to speculate that the degradation of AFB1 may be complex, involving not only oxidation by oxidases and peroxidases, but also reduction by reductases or catalysis by oxidoreductases. However, whether these enzymes have a degradative function needs to be further verified.

In general, *Bacillus megaterium* is considered to be a safe and non-toxic microorganism for agriculture and medicine use (Rios-Ruiz et al., 2023). We compared the genome of SX1-1 with the VFDB and found 248 annotated genes, which are not virulence factors but important regulators involved in the microbial growth process (Supplementary Table S3). This suggests that the strain may be safe and green. In addition to the safety of the degrading bacteria, their safety against AFB_1_ degradation products needs to be considered. Unfortunately, we do not currently have a clear resolution of their structure. Although some studies have shown that AFB_1_ can be degraded into smaller non-toxic molecules (Yue et al., 2022), further experimental validation is needed to assess the safety of AFB_1_ degradation products of AFB_1_ by SX1-1.

### 3.5 Degradation of AFB_1_-contaminated coix seeds by SX1-1

**Figure 5:**
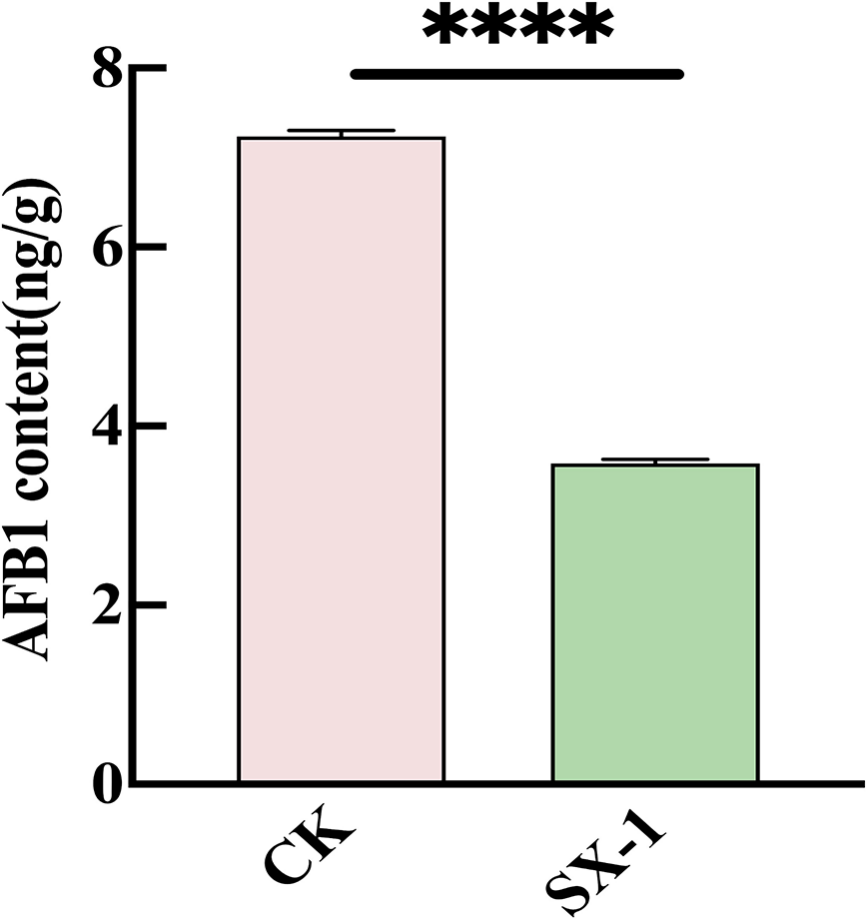
SX1-1 degradation in coix seeds *in situ* (SX1-1 supernatant co-incubated with AFB_1_ contaminated coix seeds for 72 h).

To verify the effect of SX1-1 on contaminated medicinal coix seeds, the SX1-1 supernatant was co-incubated with AFB_1_ contaminated coix seeds. After 72 h, the degradation rate was determined. The results showed that SX1-1 was able to degrade AFB_1_-contaminated coix seeds and the degradation rate on the herb was 50.06% compared to the control (Figure 5). The surface of coix seeds does not appear to have any significant changes. This suggested that SX1-1 was able to better degrade aflatoxin-contaminated coix seeds. Compared to some other strains, such as *Microbacterium protolyticum* B240 reported to degrade AFB_1_ on corn (Yan et al., 2022), the degradation of AFB_1_ in coix seed by SX1-1 was not too high, which may be due to the own quality of coix seed as well as the amount of SX1-1 added to coix seed. In addition, the enzyme activity only reaches its maximum value in a certain pH range (Xu et al., 2023a,b). When the pH-adjusted SX1-1 supernatant was added to the herbs, the change of pH might affect the degradation of SX1-1, and it is necessary to continue to explore the optimal degradation of SX1-1 in co-culture with herbs.

Nevertheless, there are potential risks associated with the use of microbial degradation and the safety of the degradation by-products needs to be fully evaluated to explain the degradation mechanism of microbial degradation of AFB_1_ and to lay the groundwork for the development of future strains. It must also be taken into account that the current amount of enzyme is insufficient for application in practical production, and that further purification and enzymatic studies of the enzyme contained in the supernatant will be needed in the future to sufficiently increase the enzyme activity. This is easier, more reproducible, more specific and more effective than using live microorganisms. Although researchers have attempted to address this issue using genetically engineered strains, the safety and efficacy of engineered bacteria remains to be thoroughly investigated.

## 4 Conclusion

This study identified a *Bacillus megaterium* SX1-1 strain that efficiently degrades aflatoxin B_1_. The site of degradation activity of this strain was the enzyme or protein of the fermentation supernatant. The optimal conditions for degradation were 72 h, 57°C and an initial pH of 8.0. In addition, based on genome sequencing, the present study showed that many potential enzymes for the degradation of AFB_1_ exist in SX1-1. And SX1-1 showed good ability to remove AFB_1_ from coix seeds. This indicates that SX1-1 is a promising strain as an emerging biodetoxifying agent for the reduction of aflatoxin in food or herbal medicines.

## Data availability statement

The datasets presented in this study can be found in online repositories. The data presented in the study are deposited in the NCBI repository, accession number SAMN40570435.

## Author contributions

TL: Writing – review & editing, Writing – original draft, Methodology, Investigation, Data curation. XC: Writing – review & editing, Writing – original draft, Methodology, Investigation, Data curation. ZQ: Writing – review & editing, Data curation. GR: Writing – review & editing, Data curation. NZ: Writing – review & editing, Data curation. JC: Writing – review & editing, Data curation. DJ: Writing – review & editing, Validation, Supervision. CL: Writing – review & editing, Validation, Supervision, Project administration.

## Supplementary material

SUPPLEMENTARY TABLE S1 Results of physiological and biochemical properties of SX1-1.

SUPPLEMENTARY TABLE S2 The enzyme of SX1-1 annotated by the COG database.

SUPPLEMENTARY TABLE S3 VFDB database prediction for SX1-1.

## References

[ref1] Arimboor R. (2024). Metabolites and degradation pathways of microbial detoxification of aflatoxins: a review. Mycot. Res. 40, 71–83. doi: 10.1007/s12550-023-00515-0, PMID: 38151634

[ref2] Bedard L. L. Massey T. E. (2006). Aflatoxin B1-induced DNA damage and its repair. Cancer Lett. 241, 174–183. doi: 10.1016/j.canlet.2005.11.018, PMID: 16458422

[ref3] Campos-Avelar I. Colas de la Noue A. Durand N. Cazals G. Martinez V. Strub C.. (2021). *Aspergillus flavus* growth inhibition and aflatoxin B-1 decontamination by streptomyces isolates and their metabolites. Toxins 13:340. doi: 10.3390/toxins13050340 34066812 PMC8151643

[ref4] Chang X.X. Jiang D. Wang J.N. Li T. Ren G.X. Mi J. (2021). Aflatoxins and changes of *Aspergillus flavus* on polygalae radix during storage. Mod. Chin. Med. 23, 1921–1926. doi: 10.13313/j.issn.1673-4890.20201022005

[ref5] Chen G. J. Shu D. Wei Z. Luo D. Yang J. Li Z.. (2022). Detoxification of aflatoxin B1 by a potential probiotic *Bacillus amyloliquefaciens* WF2020. Front. Microbiol. 13:1085000. doi: 10.3389/fmicb.2022.1085000, PMID: 35620100 PMC9127598

[ref6] Choo M. J. Hong S. Y. Chung S. H. Om A. S. (2021). Removal of aflatoxin B1 by edible mushroom-forming fungi and its mechanism. Toxins 13:668. doi: 10.3390/toxins13090668, PMID: 34564672 PMC8473272

[ref7] Deng D. Tang J. H. Liu Z. C. Tian Z. M. Song M. Cui Y. Y.. (2022). Functional characterization and whole-genome analysis of an aflatoxin-degrading *Rhodococcus pyridinivorans* strain. Biology 11:774. doi: 10.3390/biology11050774, PMID: 35625502 PMC9138218

[ref8] Fang Q. A. Du M. Chen J. Liu T. Zheng Y. Liao Z.. (2020). Degradation and detoxification of aflatoxin B1 by tea-derived *Aspergillus niger* RAF106. Toxins 12:777. doi: 10.3390/toxins12120777, PMID: 33291337 PMC7762301

[ref9] Guo C. Y. Fan L. X. Yang Q. Q. Ning M. X. Zhang B. C. Ren X. F. (2024). Impact of three exogenous phosphorus-solubilizing bacteria on zinc and selenium contents and rhizosphere soil nutrients of Longjing and Huangjinya tea plants. Front. Microbiol. 15:1413538. doi: 10.3389/fmicb.2024.1413538, PMID: 38989025 PMC11233738

[ref10] Hamad G. M. Mehany T. Simal-Gandara J. Abou-Alella S. Esua O. J. Abdel-Wahhab M. A.. (2023). A review of recent innovative strategies for controlling mycotoxins in foods. Food Control 144. doi: 10.1016/j.foodcont.2022.109350, PMID: 38815343

[ref11] Li H. B. Kang X. F. Wang S. Mo H. Z. Xu D. Zhou W.. (2021b). Early detection and monitoring for *Aspergillus flavus* contamination in maize kernels. Food Control 121:107636. doi: 10.1016/j.foodcont.2020.107636

[ref12] Li G. Li X. Dong L. Li C. Zou P. Saleemi M. K.. (2021a). Isolation, identification and characterization of *Paenibacillus pabuli* E1 to explore its aflatoxin B-1 degradation potential. Curr. Microbiol. 78, 3686–3695. doi: 10.1007/s00284-021-02624-4, PMID: 34406433

[ref13] Liu L. Xie M. Wei D. (2022). Biological detoxification of mycotoxins: current status and future advances. Int. J. Mol. Sci. 23:1064. doi: 10.3390/ijms232416220, PMID: 35162993 PMC8835436

[ref9002] Liu R. Chang M. Sun F. Wang S. Jin Q. Wang X. (2013). Optimization of solid-statefermentation removal of aflatoxin B1in peanut meal by Bacill us megaterium using response surface methodology. *China Oil Fats* 38:28–30., PMID: 36555861

[ref14] Luo S. J. Du H. L. Kebede H. Liu Y. Xing F. G. (2021). Contamination status of major mycotoxins in agricultural product and food stuff in Europe. Food Control 127:108120. doi: 10.1016/j.foodcont.2021.108120

[ref15] Liu R. Wang S. Jin Q. Wang X. (2013). Optimization of solid-state fermentation removal of aflatoxin B1 in peanut meal by *Bacillus megaterium* using response surface methodology. Chin. Oils Fats 38, 28–30.

[ref16] Pakizeh M. Nouri L. Azizi M. H. (2022). Probiotic-based optimization of pistachio paste production and detoxification of aflatoxin B1 using *Bifidobacterium lactis*. J. Food Qual. 2022, 1–31. doi: 10.1155/2022/2504482

[ref17] Qin M. Liang J. Yang D. J. Yang X. Cao P. Wang X. D.. (2021). Spatial analysis of dietary exposure of aflatoxins in peanuts and peanut oil in different areas of China. Food Res. Int. 140:15. doi: 10.1016/j.foodres.2020.109899 33648201

[ref18] Rios-Ruiz W. F. Tuanama-Reategui C. Huaman-Cordova G. Valdez-Nunez R. A. (2023). Co-Inoculation of endophytes *Bacillus siamensis* TUR07-02b and *Priestia megaterium* SMBH14-02 promotes growth in rice with low doses of nitrogen fertilizer. Plan. Theory 12:524. doi: 10.3390/plants12030524 PMC9919783 36771609

[ref19] Sun D. Mao J. Cheng L. Yang X. L. Li H. Zhang L. X.. (2021). Magnetic g-C3N4/NiFe2O4 & composite with enhanced activity on photocatalytic disinfection of *Aspergillus flavus*. Chem. Eng. J. 418:11. doi: 10.1016/j.cej.2021.129417

[ref9003] Sun F. Yu D. Zhou H. Lin H. Yan Z. Wu A. (2023). CotA laccase from Bacillus licheniformis ZOM-1 effectively degrades zearalenone, aflatoxin B1 and alternariol. Food Control 145. doi: 10.1016/j.foodcont.2022.109472

[ref20] Tang Y. Liu X. J. Dong L. He S. R. (2024). Screening and identification of an aflatoxin B_1_−degrading strain from the Qinghai-Tibet Plateau and biodegradation products analysis. Front. Microbiol. 15:1367297. doi: 10.3389/fmicb.2024.1367297, PMID: 38751722 PMC11094616

[ref21] Tatusov R. L. Fedorova N. D. Jackson J. D. Jacobs A. R. Kiryutin B. Koonin E. V.. (2003). The COG database: an updated version includes eukaryotes. BMC Bioinformatics 4. doi: 10.1186/1471-2105-4-41, PMID: 12969510 PMC222959

[ref22] Watanakij N. Visessanguan W. Petchkongkaew A. (2020). Aflatoxin B_1_−degrading activity from *Bacillus subtilis* BCC 42005 isolated from fermented cereal products. Food Additiv. Contam. Part A. Chem. Analy. Control Expos. Risk Assess. 37, 1579–1589. doi: 10.1080/19440049.2020.1778182 32723015

[ref9001] Xu Y. H. Lou H. W. Zhao R. Y. (2023). Cloning and expression of the catalase gene (KatA) from *Pseudomonas aeruginosa* and the degradation of AFB(1) by recombinant catalase. J Sci Food Agric 103, 792–798. doi: 10.1002/jsfa.12190 36054708

[ref23] Xu Y. H. Dong H. Y. Liu C. X. Lou H. W. Zhao R. Y. (2023a). Efficient Aflatoxin B1 degradation by a novel isolate, *Pseudomonas aeruginosa* M-4. Food Control 149. doi: 10.1016/j.foodcont.2023.109679

[ref24] Xu Y. H. Zhao R. Y. Liu C. X. (2023b). Degradation of aflatoxin B_1_ in moldy maize by *Pseudomonas aeruginosa* and safety evaluation of the degradation products. Food Secur. 12:1217. doi: 10.3390/foods12061217 PMC10048033 36981146

[ref25] Xue G. Qu Y. Wu D. Huang S. Che Y. Yu J.. (2023). Biodegradation of Aflatoxin B-1 in the Baijiu Brewing Process by *Bacillus cereus*. Toxins 15:1. doi: 10.3390/toxins15010065, PMID: 36668884 PMC9860622

[ref26] Yan Y. Zhang X. Y. Chen H. Y. Huang W. N. Jiang H. N. Wang C. L.. (2022). Isolation and Aflatoxin B1-Degradation Characteristics of a Microbacterium proteolyticum B204 Strain from Bovine Faeces. Toxins 14:8. doi: 10.3390/toxins14080525, PMID: 36006187 PMC9415550

[ref27] Yue X. F. Ren X. F. Fu J. Y. Wei N. Altomare C. Haidukowski M.. (2022). Characterization and mechanism of aflatoxin degradation by a novel strain of Trichoderma reesei CGMCC3.5218. Front. Microbiol. 13:1003039. doi: 10.3389/fmicb.2022.1003039, PMID: 36312918 PMC9611206

[ref28] Zhang Y. C. Wang P. Kong Q. Cotty P. J. (2021b). Biotransformation of aflatoxin B-1 by Lactobacillus helviticus FAM22155 in wheat bran by solid-state fermentation. Food Chem. 341:128180. doi: 10.1016/j.foodchem.2020.128180 33032249

[ref29] Zhang Q. Zhang Y. L. Liu S. S. Wu Y. Z. Zhou Q. Zhang Y. Z.. (2021a). Adsorption of deoxynivalenol by pillared montmorillonite. Food Chem. 343:128391. doi: 10.1016/j.foodchem.2020.128391 33268181

[ref30] Zhao Q. N. Qiu Y. Wang X. Gu Y. Y. Zhao Y. Z. Wang Y. D.. (2020). Inhibitory Effects of Eurotium cristatum on Growth and Aflatoxin B-1 Biosynthesis in Aspergillus flavus. Front. Microbiol. 11:13. doi: 10.3389/fmicb.2020.00921 32477315 PMC7242626

[ref31] Zhou H. Y. Liu N. Yan Z. Yu D. Z. Wang L. Wang K. B.. (2021). Development and validation of the one-step purification method coupled to LC-MS/MS for simultaneous determination of four aflatoxins in fermented tea. Food Chem. 354:8. doi: 10.1016/j.foodchem.2021.129497 33752112

[ref32] Zhou S. H. Yuan Q. S. Wang X. A. Jiang W. K. Ou X. H. Yang C. G.. (2023). Volatiles from *Pseudomonas palleroniana* Strain B-BH16-1 Suppress Aflatoxin Production and Growth of Aspergillus flavus on *Coix lacryma-jobi* during Storage. Toxins 15:1. doi: 10.3390/toxins15010077, PMID: 36668896 PMC9861347

