# Supplementary material for: Characterization and genomic analysis of Bacillus megaterium with the ability to degrade aflatoxin B1
Source: Front Microbiol. 2024 Aug 7;15:1407270. doi: 10.3389/fmicb.2024.1407270 (PMC11335518; doi:10.3389/fmicb.2024.1407270)
Supplement: SUPPLEMENTARY TABLE S1 — Results of physiological and biochemical properties of SX1-1. [file Data_Sheet_1.docx]

**Functional characterization and genomic analysis of *Bacillus megaterium* with the ability to degrade aflatoxin B1**

Ting Li^1^, Xiaoxi Chang^1^, Zixuan Qiao^1^, Guangxi Ren^1^, Na Zhou^1^, Jiaxin Chen^1^, Dan Jiang^1*^, Chunsheng Liu^1*^

1 School of Chinese Medicine, Beijing University of Chinese Medicine, 102488, Beijing, China

*Corresponding author: Dan Jiang ([jiangdan1027@163.com](mailto:jiangdan1027@163.com)), Chunsheng Liu ([max_liucs@263.net](mailto:max_liucs@263.net)), School of Chinese Medicine, Beijing University of Chinese Medicine, 102488, Beijing, China


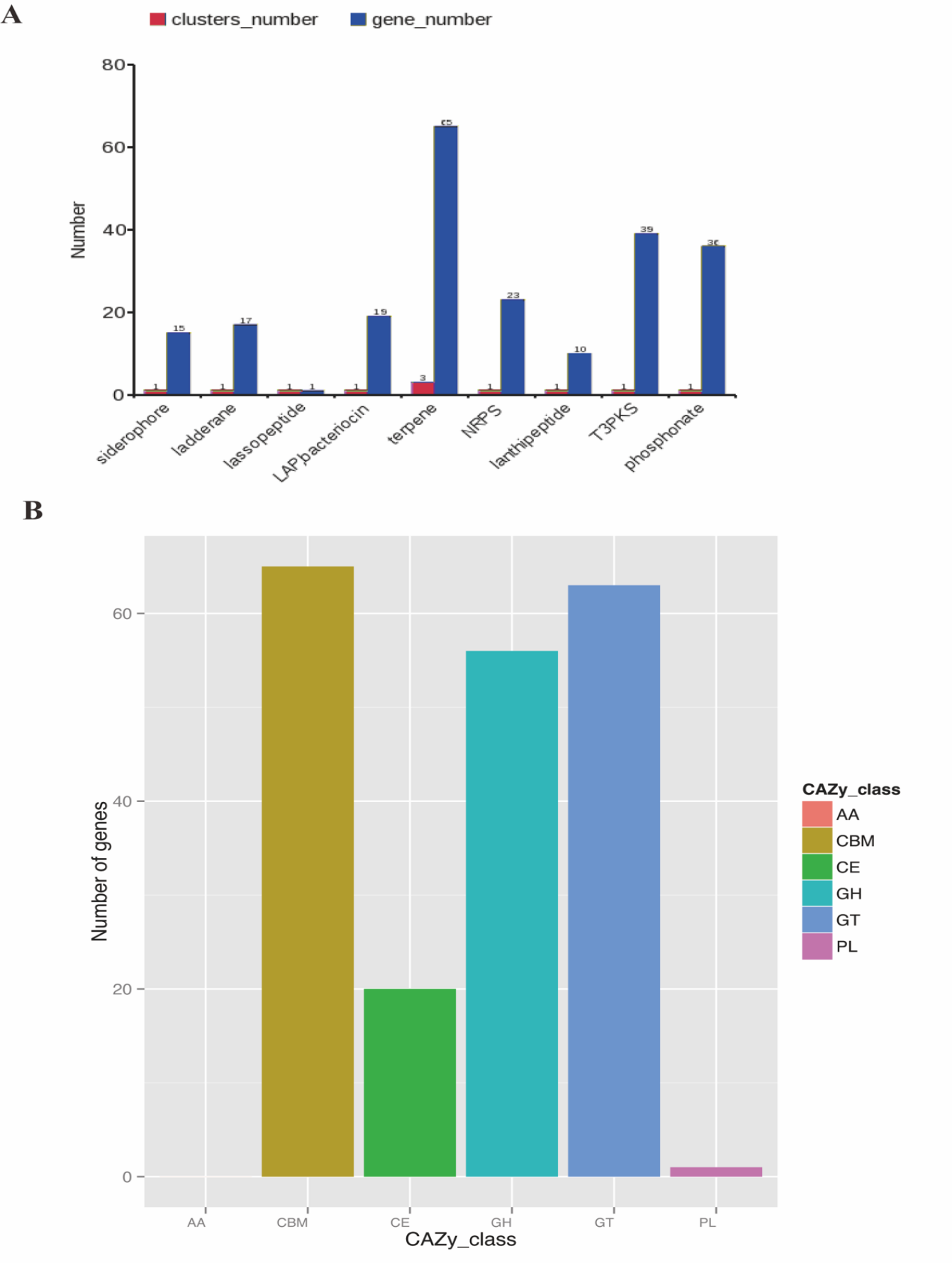


Figure S1 SX1-1 gene number classification statistics (A: Predicted SX1-1 secondary metabolite gene clusters using the antiSMASH program, red bars indicate the number of gene clusters and blue bars indicate the number of genes contained under the gene cluster. (B: functional classification of the CAZy database and analysis of the corresponding gene numbers, AA for oxidoreductase, CBM for carbohydrate-binding structural domain, CE for sugar esterase, GH for glycoside hydrolase, GT for glycosyltransferase, PL for polysaccharide lyase)

16S sequence of SX1-1

CCTCTGTCTCTTAGGCGGCTAGCTCCTTACGGTTACTCCACCGACTTCGGGTGTTA

CAAACTCTCGTGGTGTGACGGGCGGTGTGTACAAGGCCCGGGAACGTATTCACCG

CGGCATGCTGATCCGCGATTACTAGCGATTCCAGCTTCATGTAGGCGAGTTGCAG

CCTACAATCCGAACTGAGAATGGTTTTATGGGATTGGCTTGACCTCGCGGTCTTG

CAGCCCTTTGTACCATCCATTGTAGCACGTGTGTAGCCCAGGTCATAAGGGGCAT

GATGATTTGACGTCATCCCCACCTTCCTCCGGTTTGTCACCGGCAGTCACCTTAGA

GTGCCCAACTAAATGCTGGCAACTAAGATCAAGGGTTGCGCTCGTTGCGGGACTT

AACCCAACATYTCACGGACMCGAGCTGACGACAACCATGCACCACCTGTCACTY

TGTCCCCCGAAGGGGAACGCTCTATCTCTAGAGTTGTCAGAGGATGTCAAGACCT

GGTAAGGTTCTTCGCGTTGCTTCGAATTAAACCACATGCTCCACCGCTTGTGCGG

GCCCCCGTCAATTCCTTTGAGTTTCAGTCTTGCGACCGTACTCCCCAGGCGGAGTG

CTTAATGCGTTAGCTGCAGCACTAAAGGGCGGAAACCCTCTAACACTTAGCACTC

ATCGTTTACGGCGTGGACTACCAGGGTATCTAATCCTGTTTGCTCCCCACGCTTTC

GCGCCTCAGCGTCAGTTACAGACCAAAAAGCCGCCTTCGCCACTGGTGTTCCTCC

ACATCTCTACGCATTTCACCGCTACACGTGGAATTCCGCTTTTCTCTTCTGCACTC

AAGTTCCCCAGTTTCCAATGACCCTCCACGGTTGAGCCGTGGGCTTTCACATCAG

ACTTAAGAAACCGCCTGCGCGCGCTTTACGCCCAATAATTCCGGATAACGCTTGC

CACCTACGTATTACCGCGGCTGCTGGCACGTAGTTAGCCGTGGCTTTCTGGTTAG

GTACCGTCAAGGTACGAGCAGTTACTCTCGWACTTGTTCTTCCCTAACAACAGAG

TTTTACGACCCGAAAGCCTTCATCACTCACGCGGCGTTGCTCCGTCARACTTTCGT

CCATTGCGGAAGATTCCCTACTGCTGCCTCCCGTAGGAGTCTGGGCCGTGTCTCA

GTCCCAGTGTGGCCGATCACCCTCTCAGGTCGGCTATGCATCGTTGCCTTGGTGA

GCCGTTACCTCACCAACTAGCTAATGCACCGCGGGCCCATCTGTAAGTGATAGCC

GAAACCATCTTTCAATCATCTCCCATGAAGGAGAAGATCCTATCCGGTATTAGCT

TCGGTTTCCCGAAGTTATCCCAGTCTTACAGGCAGGTTGCCCACGTGTTACTCACC

CGTCCGCCGCTAACGTCATAGAAGCAAGCTTCTAATCAGTTCGCTCGACTTGCAT

GTATAGCACGCCGCCAGGTCC
